# From public vs. private to public/private mix in healthcare: lessons from the Israeli and the Spanish cases

**DOI:** 10.1186/s13584-020-00391-4

**Published:** 2020-06-24

**Authors:** Dani Filc, Alon Rasooly, Nadav Davidovitch

**Affiliations:** 1 Department of Politics and Government, Ben-Gurion University, Beersheba, Israel; 2 School of Public Health, Ben-Gurion University, Beersheba, Israel

**Keywords:** Private/public mix, Neoliberalism, Privatization, Israel, Spain

## Abstract

**Background:**

Different forms of public/private mix have become a central mode of the privatization of healthcare, in both financing and provision. The present article compares the processes of these public/private amalgams in healthcare in Spain and Israel in order to better understand current developments in the privatization of healthcare.

**Main text:**

While in both Spain and Israel combinations between the public and the private sectors have become the main forms of privatization, the concrete institutional forms differ. In Spain, these institutional forms maintain relatively clear boundaries between the private and the public sectors. In Israel, the main forms of public/private mix have blurred such boundaries: nonprofit health funds sell private insurance; public nonprofit health funds own private for-profit hospitals; and public hospitals sell private services.

**Conclusions:**

Comparison of the processes of privatization of healthcare in Spain and Israel shows their variegated characters. It reveals the active role played by national and regional state apparatuses as initiators and supporters of healthcare reforms that have adopted different forms of public/private mix. While in Israel, until recently, these processes have been perceived as mainly technical, in Spain they have created deep political rifts within both the medical community and the public. The present article contains lessons each country can learn from the other, to be adapted in each one’s local context: The failure of the Alzira model in Spain warns us of the problems of for-profit HMOs and the Israeli private private/public mix shows the risk of eroding trust in the public system, thus reinforcing market failures and inefficient medical systems.

## Background

Since the late 1970s, healthcare systems all around the world have been undergoing processes of reform, and public and academic discussions on the transformation of healthcare systems have become ubiquitous [1]. There are several reasons for these reforms, including changing therapeutic paradigms and technological developments, professional demands from medical associations, and users’ changing needs and desires. Demographic and epidemiological changes—mainly due to aging populations—have also had an important influence. However, a central reason is the transition to a global, neoliberal socioeconomic model [2–4]. During the last decade, leading medical and public healthcare figures have been raising concerns about the growing influence of neoliberalism on healthcare. This influence has been expressed in economic policies such as privatization, austerity, deregulation, free trade, and reductions in government spending on health and welfare systems. This increase in the role of the private sector in the economy and society has resulted in an ongoing process of privatization of welfare services—among them, healthcare—thus leading to growing health inequalities [5, 6].

As suggested by proponents of convergence theories, the tendency toward partial privatization of healthcare is common to most reforms. However, a comparative examination of those processes in different countries shows that the situation is more complex than described by convergence theories. While neoliberalism is pervasive, there are significant differences among countries, leaving substantial space for local context, politics, and contestation. As Brenner, Peck, and Theodor [7] argue, reform processes are simultaneously patterned by global assumptions and conditions, and are therefore both interconnected and locally specific, modulated by the institutional, historical, and political characteristics of the different countries. Understanding the tensions between the global processes and local implementation is crucial for policymakers when dealing with the consequences of recent health reforms and their influences on both efficiency and equity—two main challenges facing current healthcare systems.

The present paper compares processes of privatization of healthcare in Spain and in Israel, focusing mainly on forms of public/private combinations, to evaluate concretely the “variegated” character of neoliberalization processes. Following Brenner, Peck, and Theodor’s [7] theoretical approach, we compare the Spanish and Israeli cases as examples of the ways in which the globalization of the neoliberal socioeconomic model puts constraints on individual countries and supports processes of privatization—in the present case, of healthcare—while simultaneously increasing the uneven development of regulatory and institutional forms, an uneven development related to each country’s institutional specificities [7]. Thus, while taking cognizance of the role played by global transnational processes, this approach is also attentive to the active roles played both by national and regional state apparatuses, as initiators and supporters of neoliberal reforms, and by preexisting institutional forms.

Within the healthcare sector, privatization can be present in three interconnected, main forms. The first form is financing, meaning that an increasing share of the national expenditure in health is either financed by private insurance or is paid out-of-pocket (as copayments or wholly private expenditures). The second is healthcare provision, mostly in the form of development of privately owned services contracting with the public system, or of private services financed by private insurance. The third form is the “enterprization” of the public healthcare system; i.e., adoption of a business managerial culture by public institutions, which blurs the boundaries between the public and the private for-profit sectors [8]. Related to these three aspects, the concept of managed (or regulated) competition was introduced several decades ago, where one of the objectives was to create a balance between private and public modes of operation.

Almost by definition, the privatization of finance links the right to healthcare services to the ability to pay for them. Studies have consistently shown that when compared with countries with publicly funded universal health coverage (e.g., the UK and the Netherlands), equitable access to healthcare is severely limited in health systems where private insurance dominates (e.g., the US and Switzerland) [9–12]. In a survey that compared insurance-related experiences among adults in 11 countries during 2010 [9], 39% of US citizens with below-average income and 20% of those with above-average income experienced at least one access barrier due to cost. In the UK, on the other hand, only 4% experienced one or more access barriers due to cost, regardless of their income level. In a similar study among respondents who requested emergency care during 2016 [11], 33% of US and 22% of Swiss citizens reported experiencing cost-related access problems; whereas, only 7 and 8% had such an experience in the UK and in the Netherlands, respectively.

In support of private provision of healthcare, some researchers [13] claim that private provision allows for a more efficient utilization of resources. For example, operating room utilization in the private healthcare system is perceived as more efficient compared to such in the public healthcare system. However, numerous studies have shown that the patient composition in those two systems is entirely different [14–17]. Compared with their counterparts in the public sector, patients receiving private elective surgeries are younger, have less comorbidities, and are from a higher socioeconomic class. This widespread difference has led researchers to claim that the apparent efficiency advantage of private provision is related to “cream skimming” patients according to their risk [17–20].

Beyond the adverse outcomes concerning equity and the doubtful gains in efficiency, private provision and finance of healthcare can result in unnecessary, and sometimes harmful, cases of overtreatment. A systematic review of 21 studies [21] found that the odds of a Caesarean section (C-section) being performed was significantly higher in relation to women with private health insurance compared with women using public health insurance. In Chile, for example, three out of four publicly insured women who opt to give birth in a private hospital will have a C-section, while in public hospitals only one out of four women will undergo this procedure [22, 23]. Mixed-methods studies suggest that private obstetricians have women undergo non-medical C-sections since this procedure is more lucrative for the private practitioner and allows the “programing” (scheduling) of births [23, 24].

Evidence from Spain and Israel indicates that mixed provision of private and public services does not necessary lead to better performance, while harming equitable access and provision of health services. In Spain, the highly praised Alzira private-public partnership model was shown to perform worse than the benchmark in 15 out of 26 indicators [25]. Other studies in Spain have found that private medical insurance allows high-income individuals to avoid waiting lists and receive fast-track consultations [26, 27]. Similar implications concerning equitable access to health services were found in data relating to Israel. For instance, according to the Central Bureau of Statistics [28], 31% of Israel’s Jewish population had private insurance plans that allow access to surgical procedures in the private sector, while only 5% of that nation’s Arab population had such access in 2017. Similarly, 42% of high-income individuals had access to private specialist consultations versus 6% of low-income individuals [28]. These data emphasize how, in a mixed private-public healthcare system, the ability to pay for private insurance determines one’s access to medical procedures and consultations.

In terms of healthcare provision, within hospitals that provide both public and private medical services in Israel (this Israeli system is called Sharap, which is the Hebrew acronym for “private medical services”), a patient may be informed that a specific treatment is available in 3 months through the public system but within only a few days if s/he uses private insurance or pays out of pocket. A study conducted in two such hospitals in Jerusalem (Hadassah Hospital and Shaare Zedek Hospital) found that the average waiting times for a range of specialist appointments were 14 times longer for patients seeking a public rather than a private consultation at Hadassah Hospital ([29], p. 26). At Shaare Zedek Hospital, the waiting time was five times longer for patients seeking care through the public pathway, in comparison to the private pathway [30]. Also, private patients in hospitals with a private-public mix (Sharap) have an advantage over public patients in terms of the seniority of the lead surgeon [31]. In summary, the above studies suggest that patients receiving care through the public system in a mixed system are deprioritized in terms of access, waiting times, and seniority of the attending specialist. This implicates dimensions of social inequity as well as efficiency of resources within private-public mixed systems, since people with more severe medical conditions are less likely to receive timely care by expert specialists unless they can pay for it.

In the present paper, we seek to investigate: (a) what the main processes of privatization in healthcare in Spain and Israel are, in financing and provision of services; (b) how these processes relate to budget constraints and to neoliberal reforms; and (c) what the comparison between the Spanish and the Israeli cases can teach us about the ways to cope with both budget constraints and the effects of privatization of healthcare services. We will focus especially on the development of models of private-public mix as a central form of privatization in countries with a single-payer system.

In order to cope with growing healthcare expenditures and increasing budget constraints, many countries have adopted various models of public/private partnerships. Those models have been proposed by their supporters as the best answer to shrinking public capital investments in health [32–38]. Different forms of public/private partnerships have been implemented in order to design, finance, build, and maintain hospitals and other healthcare infrastructures [39]. In some cases, public/private partnerships also provide healthcare, whether by outsourcing or by privatizing specific services [32, 38]. While global changes in the political economy of healthcare induced the adoption of different private-public combinations in almost every country, their specific forms—the ways in which different countries adopt specific kinds of economically mixed systems of healthcare—depend on the local context.

## Main text: the comparison between Spain and Israel

Comparing the process of partial privatization of healthcare in Spain and Israel provides a good case study for evaluating neoliberal reforms and assessing the validity of the theoretical approach presented above. Healthcare reforms in Spain and Israel share similarities in their contexts, including the two countries’ levels of economic development, the characteristics of their welfare systems, and, more specifically, the development of their healthcare systems (Table 1). According to OECD 2018 data, Israel’s GDP per capita stands at $41,678, while Spain’s stands at $41,758. According to the United Nation Development Programme, the 2019 Human Development Index values for these two nations are very close (0.906 for Israel and 0.893 for Spain).

**Table 1:** Comparison between Spain and Israel

	Spain	Israel
Economic development: GDP per capita (2019)^a^	41,758$	41,678$
Human development index (2019)^b^	0.893	0.906
Health expenditure as percent of GDP (2018)^a^	8.86%	7.46%
Government/compulsory schemes as percent of all health expenditure (2018)^a^	70.47%	63.78%
Character of the state	Central state with devolution of power and responsibilities to regional autonomies	Unitary and centralized state
Parliamentarian and electoral systems	Bi-cameral, 52 provincial circumscriptions	Uni-cameral, single circumscription
Citizenship	universal but challenged by national segregationist movements	ethno-national
Characteristics of the welfare system	Similar Mediterranean regime	
Development of the health care system	Similar move from a Bismarckian, social security health care system, to a universal one, residency based access to health care	
Medical professional culture	European and identified with the welfare system	European roots, growing American influence

Sources: ^a^https://data.oecd.org/; ^b^http://hdr.undp.org/en/countries/profiles/. Accessed: May 25, 2020

Concerning the welfare regime, Spain and Israel are considered to belong to the extended family of Mediterranean welfare states [40], which includes Cyprus, Greece, Israel, Italy, Malta, Spain, Portugal, and Turkey. This Mediterranean welfare regime is characterized by relatively late industrialization, labor market rigidity and segmentation, and significant shadow economies, with implications for the protection of workers and for state revenues. Social spending in these countries, while higher than in countries with liberal welfare regimes, is lower than in social-democratic and corporatist countries, and the welfare state’s ability to overcome socioeconomic gaps is limited [40]. Among the characteristics of the Mediterranean regime are the centrality of family and religion for welfare, and the existence of universal (or near-universal) health provision by the state alongside a flourishing private healthcare market [40, 41].

Concerning specifically the healthcare system, both Spain and Israel have passed from a Bismarckian social security healthcare system to a universal one in which residency grants access to healthcare; and, in both countries, we can assess significant trends of privatization of healthcare [42–45].

Albeit these significant similarities make comparisons relevant, there are also important differences that explain Spain and Israel’s dissimilarities in their forms of private/public mix. Among these, the different character of the state (in Spain’s case, a central state has devolved power and responsibilities to regional autonomies; in Israel’s, the state is unitary and very centralized), different parliamentarian and electoral systems (Spain: bicameral, with 52 provincial circumscriptions; Israel: unicameral, with a single circumscription), different structures of citizenship (Spain: universal but challenged by national segregationist movements; Israel: ethno-national), and different medical professional cultures (Spain: more European and identified with the welfare system; Israel: more Americanized and supportive of private medicine). Thus, the combination of structural similarities—in economic development, welfare regimes, and healthcare systems—with political and cultural differences makes the comparison very useful in understanding variegated pathways of privatization.

### The Spanish case

During the last period of the Franco dictatorship, the Spanish healthcare system began to be organized following a Bismarckian model [46]. Following the transition to democracy, Article 43.1 of the 1978 constitution recognized the right of all Spaniards to health protection [47]. However, it was only in 1986, with the enactment of the Healthcare General Act (Ley General de Sanidad), that such protection was implemented within a universal healthcare system [48].

The 1986 act recognized healthcare as a fundamental right, underlining the right of equal access: Article 3.2 of that law specifically established effective equality in access to healthcare services [49]. The 1986 act gave birth to a national health system (Sistema Nacional de Salud, or SNS), with a progressive transition from payroll contributions to general taxation as the main source of financing [43, 46, 47, 50]. The SNS integrated all the functions and infrastructures that the public sector is responsible for [43, 50]. The new system covered all Spanish residents, with the exception of civil servants—the only group of people who could opt out of the national service. Civil servants are organized into three mutual funds (MUFACE, Mutualidad General de Empleados Civiles del Estado; MUGEJU, Mutual General Judicial; and ISFAS, Instituto Social del las Fuerzas Armadas) and may choose fully private provision [43, 47].

Further modifications devolved responsibility to regional authorities in a two-tiered mode. The first tier was a fast-track for regions with strong regional identities (the Basque country, Navarra, Catalonia Galicia, Valencia, Andalucia, and the Canary Islands)—some of them with self-governing traditions. The second tier, which included 10 other regions, reached autonomy in 2002 [47, 51].

The 2001 reform made funding not earmarked but, instead, part of the general sum transferred to the regions, which were responsible for decisions on how much funding they would allocate to the health budget (provided that expenditures did not fall below the 1999 sum) [47, 52]. The reform also established a new allocation formula, based on weighted capitation, taking into account population dispersion, extension, and insularity of the territory.

In Spain, thus, the development of the public healthcare system and its relations with the private sector has been a function of the interaction between the national and regional levels, with interregional differences in the degree and forms of privatization.

As a result of the 2008 economic crisis, in 2012 the Spanish government passed a royal decree (16/2012) that, in a certain way, rolled back the universal model, including in it characteristics of a Bismarckian, social security regime [43, 52, 53]. The system remained universal for emergencies; pregnancy, delivery, and post-partum care; people under age 18; and those with severe disabilities. For all other cases, the SNS provided services for those considered as insured (Royal Decree 16/2012). According to Article 3, in order to be considered as insured, a person has to fulfill one of the following conditions: be a worker covered by the social security system, be a retiree covered by the social security system, receive payments from the social security system (e.g., unemployment benefits), be a Spanish or EU citizen with an income under a certain limit (Royal Decree 16/2012). The decree also increased the scope of copayments and made them relative to income [53]. While, de facto, the Royal Decree did not modify coverage for most of the population, it made, de jure, a significant change, since the right to healthcare was no longer the basic assumption under the healthcare system [54]. The decree excluded immigrants and people 26 years and over who were not part of the labor force and therefore did not pay direct taxes [53]; it also linked entitlements to the legal and working status of individuals [43]. In July 2017, a new royal decree revoked the 2012 reform and based access on residency [55].

While the 1986 act instituted a national healthcare service financed through taxation, there was still a relatively significant private sector, with around 30% of the national health expenditure privately financed (higher than the European average) [45]. This private sector primarily included, initially, the mutual funds of civil servants insured in MUFACE, MUGEJU and ISFAS, as they could opt to receive private provision of healthcare or remain within the SSN. Some 85% of them choose the private sector [45, 56].

Concerning the private share of healthcare provision, the public system has traditionally contracted out provision of specialized care to private (mostly nonprofit) hospital providers, especially technologically sophisticated diagnostic services, or outpatient surgical procedures—many times in order to shorten wait times [43, 45, 47]. In 2014, contracts with private providers represented 12% of public health expenditures [43]. Currently, the private hospital sector represents 53% of all hospitals and 33% of all beds [45]. In 2015, private hospitals performed 29% of surgical procedures, discharged 23% of patients, and provided 23% of emergency care—figures that indicate growth in most areas of the private sector [45]. The private sector accounts for some 29% of the national health expenditure. A special case is that of Catalonia, where, due to historical reasons, two thirds of the hospital services in the SNS are provided by private nonprofit hospitals [43, 47].

During the 2000s, several hospital groups emerged in Spain, mostly financed by the private insurance sector (60% of their income comes from private insurance; 30%, from selling services to the SNS; and 10%, from private out-of-pocket [49]). These groups are partly related to transnational firms (e.g., the German Helios purchased the Quiron Group) and show a continuous tendency to concentration in large hospital groups [45]. Among the most important are the Capio Group (owning 14 hospitals), United Surgical Partners (USP; with 35 centers in Spain), the Vithas Group, and the Hospiten Group, active in the field of medical tourism [45, 48]. In the last years, the volume of private medicine in Spain reached 28.5 billion euro, representing 3.3% of the country’s GDP in 2018 [45]. Today, 30% of the workers in the health sector are employed in the private sector. Fifty thousand physicians (slightly more than a third of all physicians in Spain) practice private medicine, with a third of them working both within the public system and privately [48].

Since the 1990s and until the late 2010s, Spain has undergone a slow and partial process of privatization of healthcare. The private share of the national health expenditure has increased from 22% in 1991 to 29% in 2015 [43, 47, 57]. This is related mostly to the growth in copayments for drug prescriptions for people under 65 years, dental care, over-the-counter drugs, and optical items [47]. A second source is the increase in private insurance (though the percentage of insured population is still low in comparative terms). For-profit insurance companies provide insurance for the three abovementioned mutual funds, and they are very slowly increasing their market share among people covered by the SNS—reaching 13.4% of the population in 2011 and 16.3% in 2017 [58, 59]—with significant regional variation (in Catalonia and Madrid, over 20%) [47].

If we add those who are insured by the mutuals and choose private insurance, the percentage of people holding private insurance schemes reached 22.9% in 2015 [43]. Voluntary private health insurance is unrelated to the statutory public system; it is mostly a duplicate kind of insurance, providing coverage for the same goods and services that the public sector offers. This duplicate insurance offers greater choice, faster access to procedures and specialists, and improved amenities [47]. There are also private insurance schemes that cover services not included in the SNS, such as adult dental care. In the mid-1990s, the state implemented several reforms aimed to expand private health insurance, such as a 15% tax break applied to all private healthcare payments, replaced in 1999 by deductions for employer-purchased private insurance [47]. The role of private insurers has increased, though slowly, as a result of the 2008 economic crisis. In 2007, services provided by private insurance companies represented 7.8% of the public healthcare budget, and in 2012 that figure reached 8.8% [60].

However, the main way that healthcare has become privatized has been the development of different forms of public/private mix, a mix that “is highly relevant for explaining policy outcomes in the [Spanish] health policy sector” ([61], p. 26). Since the 1990s, several forms of such mix have developed [48, 62]. First, publicly owned foundations or institutions have come under civil (private) law, adopting organizational criteria imported from the business sector [50, 62]. Second, beginning in 1996, especially in Catalonia, “associatively based entities” (EBAs) developed; these are associations of physicians managing a health center (a form of group medical practice that sells services to the public system) [43, 62]. Third, PFIs (privately financed initiatives) expanded. This model began in Madrid in 2007 and extended to other regions, such as the Balear Islands, Castilla y Leon, and Galicia. Fourth, autonomic governments contracted with private providers for the coverage of all services within an area (the “Alzira model”) [62].

The first form, the enterprization of public healthcare institutions, began with the 1997 passage of a law that allowed for institutions within the SNS to function as private enterprises [49]. This enterprization of public institutions takes place at all levels: competition between public institutions, forms of management, role of the health professions, budget responsibilities, labor relationships (“flexibilization” of labor relationships, outsourcing of ancillary work) [37]. Thus, a constellation of new public institutions with different legal characteristics appeared: public entities, consortia, foundations, public commercial societies, autonomous organisms, and public enterprises [37].

A particular form of the enterprization of the public system (that exists also in Israel, as we show below), one that completely blurs the boundaries between public and private, is the provision of private services within public hospitals. In this model (exemplified by the Barna Clinic developed within the Hospital Clinico de Barcelona), the public hospital receives private patients, generating two queues and two levels of provision [48].

Second, the EBA model, adopted since 1996 in Catalonia, aimed to promote the passage of physicians from the public system to the private sector [37, 63, 64]. The EBAs are for-profit entities—mostly, societies with limited responsibilities but some are cooperatives—owned primarily by physicians, that sell services to the SNS. Physicians must, as a group, own at least 51% of the firm, and no single proprietor may own more than 25% of the shares (making most firms composed of at least three or four physicians). Physicians’ incomes are therefore a function of the EBA’s profits. Today, there are 13 EBAs in Catalonia, providing services to 260,000 people [65].

The third mode of private/public mix in Spain is the privately financed initiatives (PFIs). This model, including 13 hospitals in 5 Autonomous Communities, implies the development of services (mainly hospitals, but also laboratories), financed and sustained by the private sector, that receive an annual payment from the public sector for a relatively long period (20 to 30 years) [32, 43]. Among the services thus developed in Spain are hospitals in Madrid, Castilla y Leon (Burgos, Salamanca), and Barcelona, and a radiotherapy unit in the Canary Islands [48, 66, 67].

Finally, there is the Alzira model, by which the SNS pays a capitated sum to a private for-profit firm in order to develop and manage all healthcare services within a certain area, providing the area’s residents with all the services guaranteed by the SNS. The implementation of this model began in Alzira in 1997 and extended to includefive areas in Valencia and three in Madrid [43]. In the Alzira region, the community of Valencia contracted with a private group, UTE-Ribera, which is headed by the private insurance company Adeslas; it is owned by the Bank Sabadell and the Centene Corporation and financed by public regional banks (Bancaja and Cam) [38, 68]. The group was to provide a full range of services for the 250,000 inhabitants of the Alzira district. The integrated system included a university hospital, 4 health centers, and 46 primary care units. The original contract was signed for 15 years, extendible to 20, and the profit rate was capped at 7.5% annually [69]; the contract was redesigned some years after, with a higher cap on the rate of profit. Similar concessions were given in the 4 other areas, including 19% of the Autonomic Community’s population [69, 70].

In 2018, a new, autonomous government in the Valencia community did not renew the contract with Ribera Salud in Alzira; however, concessions were given in four other regions: Manises, Denia, Elche-Vinalopó, and Torrevieja [69]. It should be noted that the decision to grant concessions was based on principled motives and on difficulties in regulation, since evidence about results in terms of efficiency or access is contradictory [69]. A report by the Valencia community’s comptroller on the 2013–2016 period in the Torrevieja area found that the model was more efficient than the public system [71]. However, a thorough new study does not show conclusive differences between the model in the Alzira area and the provision of healthcare services by the public sector. The authors’ conclusion was that “this archetypical PPP has not generally outperformed public-tenured providers, although in some areas of care its developments have been outstanding” [69].

### The Israeli case

The Israeli healthcare system has always been fragmentary and complex due to the emergence of several healthcare institutions before the establishment of the Israeli State. The Ministry of Health is in charge of planning and supervision; it also runs hospitals and oversees the work of the Israeli Public Health Services, leading to conflicts of interests and difficulties in fulfilling the ministry’s planning and oversight duties. The health funds, most established before the founding of the country, are nonprofit health maintenance organizations (HMOs) responsible for the provision of health services—the “healthcare basket”—as defined by law, to their members. Until the advent of the National Health Insurance law (NHIL) in 1994, they provided healthcare services within a Bismarckian social security framework. The health funds administer and provide primary and secondary care, and finance (and sometimes provide) hospitalization services. Historically, voluntary nonprofit organizations, established before Israel became a state, have run some of the hospitals and provided emergency care. Municipalities are in charge of some preventive care and public health services, and some even run hospitals.

The Israeli case provides us with an example of both a systemic transformation (from corporatist to universal) aimed to increase equality in access, and the paradoxically rapid privatization of financing. Privatization of healthcare financing began in the early 1980s, with the decrease in government funding and the increase in out-of-pocket expenditures and members’ fees during the 1980s and early 1990s. Like Spain, Israel underwent a transformation from a social security healthcare system to a universal one. In 1994, the Knesset passed the NHIL, which organized healthcare into a universal, state-funded, system.^1^

As in the Spanish case, the new Israeli law recognized healthcare as a right, underlined the importance of equality in access to healthcare, and guaranteed a universal basket of services to every Israeli resident.^2^ The system was to be financed by an earmarked “health tax” (4.8% of income), by the (already existing) earmarked employers tax, and by the government’s general budget. The National Insurance Institute (NII) collected both the health tax and the employers’ tax, and distributed the monies among the health funds according to a weighted-capitation formula. This formula takes into account the number of members in each health fund and their age mix. (In 2010, the formula was modified to include gender, and living in the periphery. The inclusion of other indicators such as socioeconomic status and or disease severity is currently under analysis by a governmental committee of experts.) A key aspect of the NHIL was that the government would cover any difference between the funds collected by the NII and the cost of the basket of services.

The law that transformed the Israeli healthcare system into a universal single-payer ran contrary to Israel’s shift toward a neoliberal socioeconomic model, a shift that began in the mid-1980s. Thus, although the law significantly increased both equality of access and progressivity of financing, it did not take long before the process of partial privatization of healthcare—which started in the mid-1980s as part of the move to neoliberal economics—was resumed. In 1997, only 2 years after the passage of the NHIL, the government passed a Budget Reconciliation Bill that eliminated employers’ contribution to healthcare. In 1998, it passed another budget reconciliation bill—replacing the government’s commitment to bridge the gap between the cost of the health basket and the funds distributed by the NII, with the provision of a significantly lower sum to be established yearly. In order to cover the diminishing public budget, the bill introduced significant increases in copayments.

Since 1998, the government’s share of the national health expenditure has declined gradually, shifting costs to the public in the form of out-of-pocket payments or private insurance. Between 1995 and 2010, public financing of healthcare services grew 11.7%, while the private share grew 51.6% [73]. By 2014, public financing of healthcare had reached an unprecedented low of about 60%, while private spending represented 38% (see Table 2) [74, 75]. The decrease in public financing was reflected in the growth of the share of health expenditure for households. In 1997, healthcare expenditure represented 3.8% of total household expenditure; by 2001, this figure had risen to 4.9%, and in 2009, it reached 5.1%. This rise in private healthcare expenditure has had an impact on equality in access to services. Household expenditure on health was significantly higher for the more affluent 20% of the population than for the poorer 20%—by 2.9 times in 1997, increasing to 3.5 times in 2001, and 3.6 times in 2008 [76]. Compared to OECD countries, Israel has a high share of private expenditure and a low share of public expenditure [73]. Out-of-pocket payments represent some two thirds of the private expenditure (copayments, dental care and oral treatment, private doctors and private insurance, prescription drugs not included in the health basket, long-term private care). The other third goes to private insurance—roughly half of it in commercial insurance policies and the other half in private policies sold by the nonprofit health funds [77].

**Table 2:** National health expenditures in Israel by financing sector (Central Bureau of Statistics, 2014)

Year	% contribution to national expenditure	
	Government	Households
1995	75	25
1996	74.5	25.5
1999	63.7	33.6
2001	61.9	36.2
2002	63.1	34.8
2003	64.1	34
2004	63.5	34.3
2005	61.8	36.2
2006	61.6	36.3
2007	60.5	37.6
2008	61.3	36.8
2009	62	36.5
2010	60.8	37.7
2011	60.8	37.8
2012	60.8	37.6
2013	60.8	39.7

While the bulk of private expenditure is in out-of-pocket payments, the increase in private expenditure is mostly an increase in ownership of private insurance. Between 2000 and 2011, the revenues of private insurance companies grew more than fourfold, from 700 million NIS (New Israeli Shekel) to 3.1 billion NIS (Bin Nun 2013). Israel has now one of the highest private health insurance ownership rates in the world, reaching 80% of the population [78]. In 2013, the public spent 1.5 billion euros on private insurance [77].

Concerning ownership, private healthcare participation in national health expenditure rose from 18.9% in 1984 to 31% in 2013 [77, 79]. The number of private healthcare centers increased from 57 in 1980 to 185 in 2013 [79], and their share increased from 30% in 1980 to almost 50% in 2013. In areas such as nursing care, privatization has been the preferred trend, and plans for construction of new units were—and still are—focused mostly on the private sector.

As in Spain, the central method of privatization of healthcare in Israel has been the expansion of different forms of public/private mix, which, in the Israeli case, is characterized by the blurring of the boundaries between the public and private sectors. Since the 2000s, budget constraints pushed hospitals and health funds to find alternative, market-related sources of income. In order to alleviate pressure on the state budget, governments allowed the public health funds to sell private supplementary and duplicate insurance, providing for services not included in the public health basket. Hospitals developed different arrays of private initiatives in order to replace insufficient funding.

As we saw above, the private share of Israel’s health expenditures has grown mainly due to the impressive expansion of supplementary insurance sold by the nonprofit health funds, from 49% of the population in 1999 to 75% in 2011 [78, 80], rates that have continued to increase but at a slower pace during the last decade. Supplementary insurance covers services not included within the public health basket, such as certain diagnostic procedures and pharmaceuticals. It also covers alternative and cosmetic medicines. However, the main reason drawing people to buy this kind of insurance policy is that it allows them to choose a specialist and to skip queues [42]. These programs allow one to choose his/her surgeon for procedures performed in private (and in some public) hospitals, and, thus, although not their original objective, they are used to shorten wait times.^3^ Moreover, the public sick funds own private for-profit hospitals, medical imaging, and laboratory facilities.

During the 2000s, many of the government-owned facilities were transformed into trusts; that is, into businesslike institutions. Government-owned public hospitals were required to behave as business firms and “sell” their “products” at full market price. Moreover, public hospitals use the existing infrastructure in order to expand services beyond their regular ones. Public hospitals run private services, such as institutes for plastic surgery. They also provide services not included in the public health basket to patients insured by commercial insurance companies (check-ups, certain laboratory tests, “personalized medicine,” etc.). This transformation of government-owned public hospitals into “market producers” has been a process that has taken place over the last 15 years. As estimated by a former deputy general director of the Ministry of Health, by the late 1990s, the health basket determined 90% of hospital activities, and hospitals “sold” the other 10% [81]. Between 1994 and 1996, the Ministry of Health allowed public hospitals to sell private services of up to 20% of their income [82]. Even though this process had already started before the enactment of the NHIL, during the last two decades its scope has expanded, from private lodging for women giving birth to medical tourism [83].

The hospitals incorporated these private and semi-private initiatives into their routine activities via three main instruments: Sharap (Hebrew acronym for “private medical services”), Sharan (Hebrew acronym for “additional medical services”), and the operation of private facilities within the public hospitals. Sharap is a system by which patients may choose their physician in a public hospital by paying an additional fee. It was implemented at the Hadassah Medical Center in the 1950s but forbidden in government-owned hospitals by the attorney general in 2002, a decision upheld by the Supreme Court in 2009 (*Kyriati* ruling; see also [84]). Under the Sharan system, public hospitals sell services not covered by the health funds—either to private insurers or to individuals. Even the main example of PFIs (privately financed initiatives) in Israel is characterized by the blurred boundaries between private and public. As one example, a new hospital has been built in the city of Ashdod by Assuta Medical Centers, a private for-profit corporation owned by the nonprofit Maccabi health fund. Assuta and the state will jointly finance the costs, and the new hospital had planned to dedicate 25% of its activity to private patients (using the Sharap arrangement)—an agreement that was finally cancelled [78].

The ongoing reforms have created a tiered system that differentiates between those who have only public insurance, those holding insurance schemes sold by the public health funds, and those holding private insurance [85]. Furthermore, most of the new private services within the public system are provided in the country’s central area (around Tel Aviv and Jerusalem), increasing existing inequalities in service provision between the center and the periphery [86]. Thirdly, the public/private mix is less efficient, as shown by its higher loss-ratio and the increase in patients shopping for third and fourth opinions [87]. Finally, another consequence of the tiered system has been the erosion of citizens’ trust in the public sector [88, 89].

### Public/private mix in Spain and Israel

We can see that the Spanish and Israeli cases have some salient similarities, and some significant differences. First, both countries underwent a transition from a Bismarckian model to a universal health system during a period in which neoliberal globalization had already begun and in which both countries had already initiated their evolution to neoliberalism. In both nations, the relevant legislation (in Spain in 1986 and in Israel in 1994) recognized healthcare as a right and stressed the centrality of equal access to healthcare. Secondly, and in apparent contradiction with the universalization of the system, both countries underwent processes of partial privatization of healthcare at three different levels: privatization of financing, privatization of ownership, and the enterprization of the public system. Moreover, in both countries the public/private mix represents a central, if not *the* central, form of privatization, and experts see it as a main threat to the future of the public healthcare system [48, 61, 62, 83].

While these similarities are striking, there are some significant differences. From the beginning, the two countries implemented their universal system in different ways. In Spain, there was a combination of a national organization with progressive devolution to the autonomous regions; in Israel, universalization was implemented through the health funds, which were the central institutions of the Bismarckian model. These different institutional forms, as shown below, have resulted in different forms of privatization and, chiefly, in different forms of private/public mixes.

**Fig. 1:**
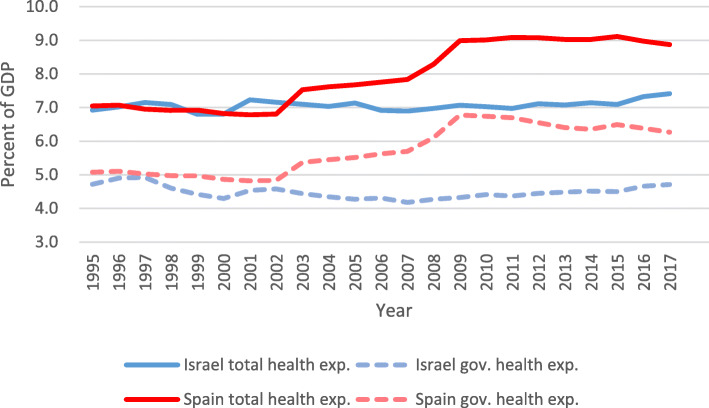
Health expenditure in Israel and Spain as percent of GDP (1995–2017). Source: OECD health statistics. Data extracted on December 2019 from OECD.Stat

The partial privatization of finance is greater in the Israeli case than in the Spanish one. Figure 1 (below) shows that, in Spain, public health expenditure as a percentage of the GDP is higher than in Israel. Moreover, while in Spain the public’s share of national health expenditure went down from 75% in 1995 to 71% in 2012, in Israel it went down from 70% in 1995, to 60.4% in 2012 [47, 57, 74]. In both countries, the number of people owning private healthcare insurance grew. However, in Spain those figures are still low (13.4%), while in Israel such growth has been exponential; today, 80% of the Israeli population owns private insurance [58]. This, even though Spain offers economic incentives for purchasing private insurance (in the form of tax exemptions), and Israel does not. While the effect of these incentives may be small in terms of size and target group, one would expect at least a smaller difference in ownership of private insurance between Israel and Spain.

Regarding privatization of ownership, both countries have seen an increase in private ownership of healthcare facilities, but in Spain the penetration of transnational firms has been much more significant. In Israel, private groups are local, and there are no significant investments of foreign capital in the healthcare sector. Finally, even though forms of private/public mix are central to the processes of privatization in both countries, the concrete institutional forms differ. Spain has three main forms of public/private mix: outsourcing of services (as in Catalonia), PFIs (as in Madrid), and the Alzira model (in the Valencia region). These mixes have taken different forms in the different autonomous regions but, in general, without blurring the boundaries between the private and the public healthcare sectors (with some exceptions, as the abovementioned hospital in Barcelona). The blurring of boundaries between private and public, in Spain, has occurred outside the healthcare system; for example, with public regional banks providing financing for the private consortium running the Alzira project. In Israel, on the other hand, there is a certain amount of outsourcing of services, but the main forms of private/public mix blur the boundaries between the public and the private healthcare system: the public health funds sell private insurance, public nonprofit health funds own private for-profit hospitals, and public hospitals sell private services.

This comparison of the processes of healthcare privatization in Spain and Israel shows the variegated character of neoliberalization—the ways in which the global transition to a neoliberal model does not result in convergence but in the “systemic production of geoinstitutional differentiation” [7]. From this perspective, processes are “polymorphic, interscalar *constructions*—born of transnational, national and (newly devolved) subnational institutional reform frameworks” ([7], p. 196). Moreover, these processes are not linear and unidirectional; they create “hybrid” institutional forms and policies “in which commodifying *and* market-constraining logics commingle and co-evolve” ([7], p. 189). In comparing the Spanish and the Israeli cases, we can see how global processes (modifications in the modes of production, capital mobility, deregulation, the strength of the neoliberal paradigm) combine with specific transnational processes (EU and euro constraints in the Spanish case; consequences of a prolonged conflict, in the Israeli one), and with national and subnational institutional and cultural characteristics. The latter are important, because they explain the differences in the ways privatization takes place in both countries. In Spain, the dynamics between the national government and the regional autonomies are central, with different regions adopting different public/private mixes (differences between Madrid, Catalonia, and Valencia, as noted above, or between Catalonia and Andalucia, as reported in [61]). Moreover, the firm opposition to privatization of the health professions working in the public sector [53] did not allow for the blurring of the boundaries between private and public.

In Israel, the health funds’ historical role and the public hospitals’ institutional strength and relative independence are the reasons that the main forms of private/public mix—private insurance schemes owned by the public health funds, private services provided by public hospitals—took place within those institutions. Moreover, because of increasing support for public/private modes of healthcare delivery among physicians working within the public sector (combined with general public opposition to the privatization of healthcare), private/public mixes that blurred the boundaries between the public and private sectors became an easier—thus preferable—path. While, in Israel, these processes were quite consensual among the medical profession, which perceived them as mainly technical, in Spain privatization of healthcare is perceived as a much more political issue, and resistance has emerged from some parts of the medical profession, as well from the public.

## Conclusions

The comparison between the Spanish and Israeli cases confirm Brenner et al.’s [7] claim that reforms are the uneven and unstable result of the influence of transnational and national forces on preexisting institutional forms, which provide fields of opportunity and spaces of realization for the neoliberazation processes. On one side, we see the penetration of market forces, the partial privatization of finance, the enterprization of the public system: all local responses to constraints imposed by the global neoliberal model. On the other side, the institutional differences between both countries show that processes of neoliberalization are constitutively uneven in terms both of institutions and of forms of regulation.

The comparison shows that those processes are not only nonlinear—as exemplified by the institutionalization in both countries of an equal right to healthcare even while in the neoliberal age—but also not unidirectional (from the global level to the national). The analysis of the Spanish and Israeli cases shows the active role played by national and regional state apparatuses as initiators and supporters of neoliberal reforms. In the Spanish instance, we see the modifications in legislation that allowed for the enterprization of the public system, the adoption of PFIs by the Madrid community, the adoption of the Alzira model at the regional level, and, under the 2012 Royal Decree, the symbolic abandonment of the universal model as a response to the economic crisis of 2008. Spain coped with budget constraints resulting from neoliberal policies, medical advances, and demographic changes by restricting the scope of healthcare coverage and allowing autonomous regions to move to different forms of public/private mix. Israel coped by decreasing the public share of the national health expenditure while blurring the boundaries between public and private—for example, the provision of private insurance schemes by the public funds. In Israel, we see neoliberal reforms in the enterprization of the public system as local responses to budget constraints, the complete blurring of the boundaries between private and public, the exponential growth in private insurance due to its marketing by the public health funds.

While the rapid growth of privatization in healthcare provision has grown rapidly, the health-policy community in both countries are scrutinizing the models of mixed-financing provision. This has resulted, in some cases, in acts of resistance toward several private/public partnerships or in the rejection of elements that increase inequity in care within those partnerships. For example, as numerous administrative and financial doubts emerged regarding Spain’s Alzira model of private/public partnership, in 2018 Valencia’s Health Authority decided to terminate the concession and to revert to direct public provision of healthcare [25]. At the national level, Spain’s socialist government approved a new Royal Decree in 2018 that made access to healthcare universal once more, including for undocumented migrants, thus abolishing the 2012 decree.

In Israel, the Ministries of Finance and Health implemented measures aimed to limit the obscuring of boundaries between the public and private sectors. In 2014, the MOH (Ministry of Health) Committee for Strengthening the Public Healthcare System decided not to expand the Sharap system to public hospitals outside Jerusalem [90]. Moreover, the state reverted previous governmental decisions that resulted in the blurring of boundaries between public and private. For example, the Assuta hospital network (Israel’s biggest private-hospital network) built a hospital in Ashdod that would provide both private and public services. In 2016, the MOH decided to compensate the network in exchange for its cancelling the provision of private services [91]. This agreement was reached following several years of judicial petitions against the expansion of the Sharap private-provision model to additional hospitals in Israel. The MOH also aims to limit the ways in which physicians blur the frontiers between public and private—for example, establishing that physicians will not be able to privately treat patients they had seen in the public sector during the previous 6 months.

These examples show that in Spain revisions of previous policies took place mostly at the macro level, while in Israel they took place at the meso, or even micro, level. While these different approaches may be related to the fact that in Israel payer competition exists between the funds but in Spain, it does not; still, each country may learn from the other, since combinations of macro-, meso-, and micro-level policies should be the best strategy to strengthen the public healthcare system. Any such strategy, though, as policymakers in both countries recognize, requires increasing public funding. Hence, the socialist government in Spain committed to raise public spending in health from 6 to 7% of GDP, until 2023 [92], and the Israeli health community demanded 15 billion NIS in order to maintain the quality of its public healthcare system.

A comparative perspective is important, since countries can learn from each other. The Spanish case offers two main teachings for Israeli healthcare policymakers, planners, and managers. The first is that the failure of the Alzira model—that is, the gap between the initial proposal and the real costs [70], lower performance when compared with public tenures [25], and difficulties in supervising services [68, 70]—warns us of the problems of for-profit HMOs. The warning is of importance, since both the MOF (Ministry of Finance) and the MOH have seriously considered the idea of allowing a for-profit HMO in Israel to compete with, currently, the four public ones.

The second teaching concerns the importance of cultural factors, and not only economic considerations, in citizens’ decisions related to healthcare. While Spain provided economic incentives for the acquisition of private insurance packages through tax exemptions, Israel did not. However, as shown above, the percentage of the population having private insurance schemes is much higher in Israel than in Spain. The low cost of those schemes in Israel may partially explain this fact. However, in order to fully understand it we have to take into account such cultural issues as confidence in the public system or attitudes toward uncertainty [89]. The Israeli case offers three main lessons for Spanish healthcare policymakers, planners, and managers. First, solutions using a private/public mix risk eroding trust in the public system, thus reinforcing market failures and inefficient medical systems (for example, by shopping for third and fourth opinions) ([29], p. 49]. Second, growing privatization in the form of supplementary insurance creates less-efficient systems. This is expressed in greater loss-ratios and in the fact that the sick funds’ private-insurance schemes create a way to bypass the efficient decision-making process of the public healthcare basket [87]. Finally, public/private mix forms (such as Sharap) show that, in fact, the public system subsidizes private users, as in the case of the Hadassah hospital, where private surgical interventions were performed all day. Thus, public facilities serve private patients at the expense of those in the public sector [29].

The shared experience of Israel and Spain with privatization of health services shows that members from the health-policy community as well as civil-society activists can spearhead a reevaluation of models of private/public mix and, in some cases, change the trajectory from private toward public provision of services.

## Data Availability

Data sharing not applicable to this article as no datasets were generated or analyzed during the current study.

## Abbreviations

EBA: Entidades de Base Asociativas
HMO: Health Maintenance Organization
ISFAS: Instituto Social del las Fuerzas Armadas
MOH: Ministry of Health
MOF: Ministry of Finance
MUFACE: Mutualidad General de Empleados Civiles del Estado
MUGEJU: Mutual General Judicial
NHIL: National Health Insurance law
NII: National Insurance Institute
NIS: New Israeli Shekel
OECD: Organization for Economic Cooperation and Development
PFI: Privately financed initiatives
SHARAN: Additional medical services
SHARAP: Private medical services
SNS: Sistema Nacional de Salud
USP: United Surgical Partners

## Footnotes

1 Addressing the reasons for the apparently paradoxical transition to a universal system during a period of neoliberal reforms exceeds the scope of the present paper. For possible explanations, see [72].

2 However, unlike as in Spain, the system is open only for “legally” recognized residents.

3 It should be noted that supplementary insurance schemes sold by the public sick funds are community rated and have no underwriting (see, Chernichovsky 2013 [73]).
